# Chondroprotection in Models of Cartilage Injury by Raising the Temperature and Osmolarity of Irrigation Solutions

**DOI:** 10.1177/1947603516688511

**Published:** 2017-01-30

**Authors:** Noha M. Eltawil, Saima Ahmed, Luke H. Chan, A. Hamish R. W. Simpson, Andrew C. Hall

**Affiliations:** 1 Centre for Integrative Physiology, Deanery of Biomedical Sciences, University of Edinburgh, Edinburgh, UK; 2 Department of Orthopaedics and Trauma, Royal Infirmary of Edinburgh and University of Edinburgh, 49 Little France Crescent, Edinburgh, UK

**Keywords:** cartilage, injury, chondroprotection, chondrocyte, viability, irrigation

## Abstract

**Objectives:**

During arthroscopic or open joint surgery, articular cartilage may be subjected to mechanical insults by accident or design. These may lead to chondrocyte death, cartilage breakdown and posttraumatic osteoarthritis. We have shown that increasing osmolarity of routinely used normal saline protected chondrocytes against injuries that may occur during orthopedic surgery. Often several liters of irrigation fluid are used during an orthopedic procedure, which is usually kept at room temperature, but is sometimes chilled. Here, we compared the effect of normal and hyperosmolar saline solution at different temperatures on chondrocyte viability following cartilage injury using *in vitro* and *in vivo* models of scalpel-induced injury.

**Design:**

Cartilage injury was induced in bovine osteochondral explants and the patellar groove of rats *in vivo* by a single pass of a scalpel blade in the presence of normal saline (300 mOsm) or hyperosmolar saline solution (600 mOsm, sucrose addition) at 4°C, 21°C, or 37°C. Chondrocytes were fluorescently labeled and visualized by confocal microscopy to assess cell death.

**Results:**

Hyperosmolar saline reduced scalpel-induced chondrocyte death in both bovine and rat cartilage by ~50% at all temperatures studied (4°C, 21°C, 37°C; *P* < 0.05). Raising temperature of both irrigation solutions to 37°C reduced scalpel-induced cell death (*P* < 0.05).

**Conclusions:**

Increasing the osmolarity of normal saline and raising the temperature of the irrigation solutions to 37°C reduced chondrocyte death associated with scalpel-induced injury in both *in vitro* and *in vivo* cartilage injury models. A hyperosmolar saline irrigation solution at 37°C may protect cartilage by decreasing the risk of chondrocyte death during mechanical injury.

## Introduction

Articular cartilage has an extraordinary durability and capacity to adapt to the physiological loading patterns associated with normal joint activities.^1^ However, exposing the tissue to unphysiological mechanical insults can result in chondrocyte death, potentially rendering articular cartilage more vulnerable to degeneration and development of posttraumatic osteoarthritis (PTOA).^2^ Arthroscopic and open surgical interventions on articular cartilage may subject the tissue to various iatrogenic injuries resulting from cutting, probing, or drilling.^3,4^ Such injuries may be associated with loss of chondrocyte viability, cartilage damage, and poor lateral integration and therefore potentially affect the surgical outcome.^5^ Minimizing chondrocyte death resulting from iatrogenic surgical injuries would thus be beneficial in reducing cartilage damage, promote the repair response, and improve integrative healing and patient outcome.^5^

Various irrigation solutions are used in orthopedic surgical practice to improve visualization of the joint space and cartilage surface. During this process, the synovial fluid is replaced with an artificial solution that has a lower osmotic pressure (~250-300 mOsm) compared with that of normal synovial fluid (~400 mOsm).^6^ Therefore, chondrocytes are exposed to a marked decrease in extracellular osmolarity during articular surgery, which is likely to affect their response to injury.^7^ For example, lowering the osmolarity of the irrigation solution increased cell death caused by mechanical cartilage injury however raising osmolarity provided significant chondroprotection against mechanical trauma caused by drilling,^4^ impact,^7^ or scalpel cutting.^8^ Using an *in vivo* model of scalpel-induced cartilage injury, we have shown that irrigating the joint with hyperosmotic saline solution significantly decreased the extent of chondrocyte death and promoted a cartilage repair response with no harmful effect on other joint tissues.^9^ Of interest is the observation that in an *in vitro* cartilage injury model at the site of the scalpel wound, the abnormal morphology of chondrocytes may be normalized by hyperosmolarity.^10^ In addition, protection of chondrocytes against the exposure of cartilage to static^11^ or moving air^12^ has been demonstrated using various hydrating solutions.

Temperature is an important factor that affects cell viability and metabolism^13,14^; however, the optimum temperature of the irrigation solution used in joint surgery has not yet been reported. Often a high volume (multiple liters) of irrigation fluid is used during an arthroscopic procedure, this is usually kept at room temperature, but is sometimes chilled as cold solutions have been proposed to reduce postoperative pain and inflammation after arthroscopic surgery.^15^ However, no statistically or clinically significant effect was found in the first 4 postoperative days.^16^ Moreover, an *in vivo* study investigating the ultrastructural surface changes of articular cartilage in rat knee joints using scanning electron microscopy, showed an uneven cartilage surface and fibrillation in joints irrigated by saline solution at 4°C and a normal, even, cartilage surface in joints flushed with saline at 37°C.^17^ It has also been demonstrated that chondrocyte metabolism and RNA synthesis were suppressed on exposure to cold saline solution.^14^

The aim of this study was therefore to investigate the effect of a normal and hyperosmotic (potentially chondroprotective) saline solution at different temperatures on chondrocyte viability in carefully controlled *in vitro* and *in vivo* scalpel-induced models of cartilage injury.

## Materials and methods

### In Vitro Cartilage Injury Model

**Figure 1.**
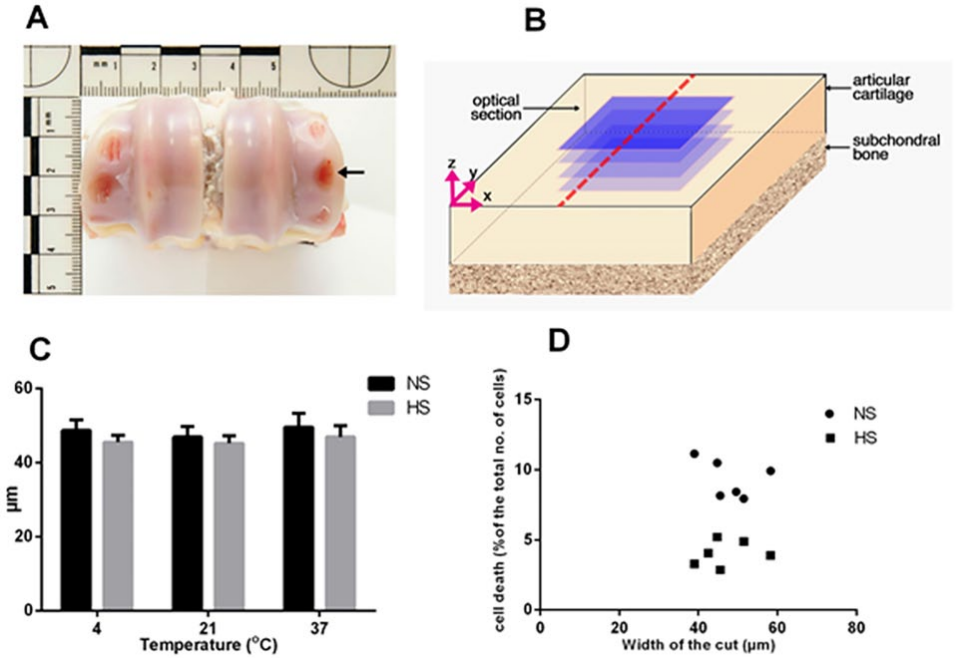
Generation and validation of an *in vitro* model of scalpel injury. (**A**) Bovine metacarpophalangeal joint with areas of exposed bone where osteochondral explants were removed (arrow). (**B**) Schematic drawing of the scalpel cut (broken red line) and the optical sections in blue, each acquired at 10-µm intervals. (**C**) Measurement of the cut width in osteochondral bovine explants exposed to normal saline solution (NS) or hyperosmolar saline solution (HS) at the indicated temperatures. Data are expressed as mean ± SEM, [6(36)]. (**D**) Scatterplots of percentage cell death in different explants against the width of the scalpel-induced cut in bovine osteochondral explants irrigated with either normal saline or hyperosmotic saline at 21°C.

Bovine osteochondral strips were harvested from the metacarpophalangeal joints of 3-year old cows (**Fig. 1A**) under aseptic conditions. These were then trimmed to produce rectangular explants of approx. 5 mm × 4 mm,^8^ immediately placed in serum-free Dulbecco’s modified Eagle’s medium (DMEM, Invitrogen, Paisley, UK) and used within 30 minutes. The serum-free medium was used to provide more controlled environment for the cells and to eliminate the variance that may occur due to variability of serum composition. The osteochondral explants were immersed for 5 minutes in either normal saline (NaCl 300 mOsm, Baxter Healthcare Ltd, Berkshire, UK) or hyperosmolar saline solution (600 mOsm, 105 g sucrose addition to 1 L normal saline)^8,9^ and maintained at 4°C, 21°C, or 37°C. In this study, sucrose was the preferred osmolyte as it is impermeable, not metabolised by chondrocytes, relatively benign and thus one experimental variable. The addition of NaCl to raise osmolarity on the other hand can potentially alter a range of processes including the activity of sodium-dependent transport processes and membrane potential, and thus it would be difficult to separate out the effects of osmolarity from sodium-dependent effects.^9^

The osmolarity of the solutions was measured by freezing point osmometer (Vitech Scientific Ltd, Horsham, UK). Explants were wounded with fresh No. 11 scalpel blades to produce a single partial thickness longitudinal cut in the middle of explant (**Fig. 1B**) and then returned to the designated solution for a further 5 minutes after injury before being assessed for cell viability.^8^ Control uninjured explants were exposed to normal or hyperosmolar saline solution at 4°C, 21°C, or 37°C for 10 minutes before staining and fixation for microscopic analysis to determine the effect of raised osmolarity and temperature of the irrigation solution on chondrocyte viability.

### In Vivo Scalpel Injury Model

Injury to the rat knee joint cartilage was induced as previously described.^9^ Briefly, male Sprague-Dawley rats (8 weeks old) were anesthetized using 3% isoflurane. The patella was dislocated laterally after medial parapatellar arthrotomy to expose the patellar groove. The articular cartilage was then wounded along the groove by a single pass of a fresh No. 11 scalpel blade held in a standard holder, and applied under its own weight. Joints were irrigated for 5 minutes before, and 5 minutes after the induction of the cartilage injury by normal saline (300 mOsm) or hyperosmolar saline solution (600 mOsm) at 4°C, 21°C, or 37°C to allow the *in situ* chondrocytes to respond to altered osmolarity^18^ (6 joints from separate animals were used in each group). Note that the open joint area was irrigated with the test solutions, and that a true lavage involving the closed joint with fluid applied under pressure was not performed. The patella was then relocated and the wound sutured in layers with coated vicryl 6-0 (polyglactin 910, Ethicon, Livingston, UK). Animals were sacrificed immediately after surgery, and knee joints were dissected and assessed for chondrocyte viability. Contralateral joints were subjected to arthrotomy and patellar dislocation without inducing cartilage injury (sham-operated) and irrigated with either normal or hyperosmolar saline solution at 4°C, 21°C, or 37°C for 10 minutes before dissection and staining. All procedures were approved by the Local Ethics Committee and Animals (Scientific Procedures) Act 1986 UK Home Office.

### Cell Viability Assay

*In situ* chondrocyte viability was determined by standard methods.^8,9^ Briefly, bovine osteochondral explants or rat knee joints were incubated with 5-chloromethyl-fluorescein diacetate (CMFDA) and propidium iodide (PI) (1 hour; both 10 µmol/L, Invitrogen, Paisley, UK) to label live/dead cells respectively. Following labeling, samples were fixed in 4% formalin (Fisher Scientific, Loughborough, UK) overnight and then stored in phosphate buffered saline (PBS) at 4°C prior to confocal imaging.

### Cartilage Imaging by Confocal Laser Scanning Microscopy

Fluorescently labeled and fixed osteochondral explants and rat knee joints were anchored to the base of a Petri dish with Blu-Tack (Bostik Ltd, Leicester, UK), immersed in PBS with the articular surface facing upward. Consecutive series of axial optical sections of the labeled *in situ* chondrocytes were acquired using a Zeiss Axioskop LSM510 confocal laser scanning microscope (CLSM; Carl Zeiss Ltd, Cambridge, UK) at 10-µm intervals (**Fig. 1B**). A standard multitrack protocol involving excitation by argon (excitation wavelength = 488 nm) and helium-neon (excitation wavelength = 543 nm) lasers, with band-pass filters (500-550 nm) and long-pass filters (>560 nm) was used to detect fluorescence emitted from CMFDA and PI and therefore allow the visualization of live and dead cells, respectively, within each optical section. Laser power, detector gain, and sensitivity were manually adjusted before acquiring each image to capture the optimal signal from each section without pixel saturation. A 3-dimensional reconstruction of all optical sections was then created using imaging software (Velocity 4·0, UK). The region of interest (ROI) applied for the *in vivo* experiments was 200 µm from the center of the scalpel injury (*x*-axis) × 921 µm (*y*-axis) × 40 µm (*z*-axis), whereas for the *in vitro* experiments the ROI was 150 × 921 × 60 µm. The quantification of *in situ* chondrocyte death after injury was then determined within these ROIs (**Fig. 1B**).

Live and dead cells were identified in the green and red channels, respectively, by thresholding voxel (volumetric pixel) intensity. A histogram of measured values for all objects in each channel was used to set the percentage threshold for the intensity (upper limit =100%; lower limit >5%) and the percentage cell death (PCD = 100 × number of dead cells/number of dead and live cells) calculated in the ROI. The width of the injury was measured by LSM imaging software (Carl Zeiss Ltd) at 100-µm intervals along the *y*-axis using the CLSM images. In all samples, 10 widths per projection were taken to calculate the mean width.

### Statistical Analysis

Data analyses were performed using GraphPad Prism (version 6.0, GraphPad Software, Inc., San Diego, CA, USA). All data were presented as mean ± standard error of mean (SEM) where *N* refers to the number of individual knee joints analyzed from separate animals and *n* to the total number of bovine explants with data shown as [*N*(*n*)]. Pearson product-moment correlation was used to test the relation between the cut width and cell death and 1-way analysis of variance (ANOVA) followed by a *post hoc* Holm Sidak correction were used to compare normal or hyperosmolar saline solution groups across all temperatures. Differences were considered statistically significant when *P* < 0.05.

## Results

### Assessment of Chondrocyte Viability Following Exposure to Varying Osmolarities and Temperatures

**Figure 2.**
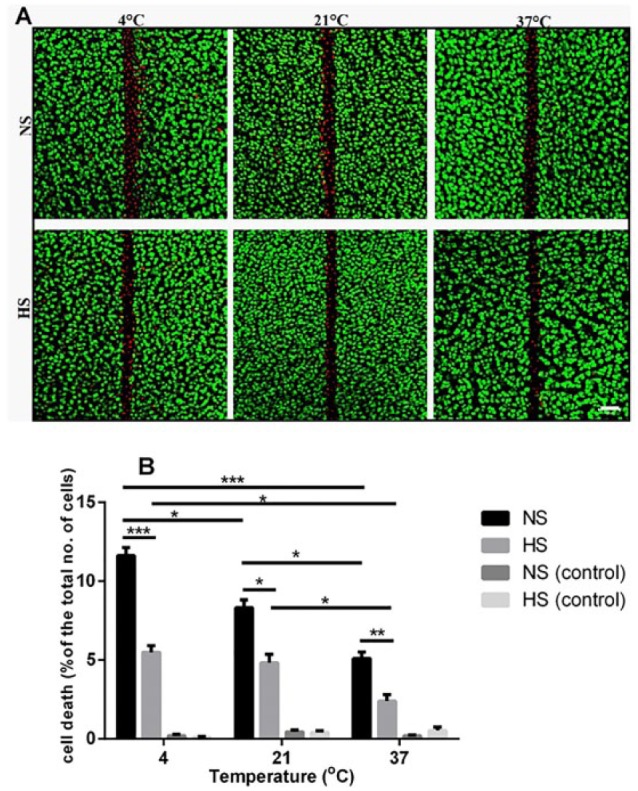
Effect of the hyperosmolar saline solution on chondrocyte death in injured osteochondral explants at different temperatures *in vitro*. (**A**) Axial confocal laser scanning microscopy (CLSM) reconstructions of 5-chloromethyl-fluorescein diacetate (CMFDA) and propidium iodode (PI) labeled chondrocytes in injured bovine explants exposed to normal saline solution (NS) or hyperosmolar saline solution (HS) at the indicated temperatures. Scale bar = 50 µm. (**B**) Pooled data for percentage cell death of explants exposed to normal saline (NS) or hyperosmolar saline solution (HS) at 4°C, 21°C, or 37°C. Data are expressed as mean ± SEM, [5(30)] for each data set. *P < 0.05, **P < 0.01, ***P < 0.001.

Control uninjured bovine osteochondral explants and sham-operated rat knee joints were irrigated with either normal or hyperosmolar saline solution at 4°C, 21°C, or 37°C to evaluate the effect of increasing osmolarity and temperature on cell viability. The PCD in articular cartilage was negligible (<1%) under all conditions in both *in vitro* and *in vivo* experiments (see **Fig. 2B**).

### Development of an In Vitro Cartilage Injury Model

Cartilage injury in osteochondral bovine explants was induced by fresh scalpel blades to produce a single longitudinal partial thickness cut in the middle of explant (**Fig. 1A** and **B**). To assess the reproducibility of the cut across all the experimental conditions, the mean width was calculated from 10 measurements taken at 100-μm intervals. The width of the defect was 47.1 ± 6.5 µm (mean ± SD; *n* = 36) with no significant difference between the groups (*P* > 0.05; **Fig. 1C**). Within this range, there was no significant correlation between the cut width and cell death (*P* > 0.05; **Fig. 1D**) for samples irrigated in either normal or hyperosmolar saline. This suggested that this standardized injury produced a reproducible zone of cell death and therefore would allow further assessment of the effect of solution osmolarity and temperature on chondrocyte viability.

### Hyperosmolarity and Physiological Temperature Reduce Chondrocyte Death due to Scalpel-Induced Injury in Bovine Cartilage

Axial CLSM projection images showed a band of cell death at/around the edges of scalpel-induced injury in bovine osteochondral explants (**Fig. 2A**). Exposing cartilage explants to hyperosmolar saline solution significantly (*P* < 0.001, *P* < 0.05, *P* < 0.01 for 4°C, 21°C, and 37°C, respectively) reduced cell death by approximately 50% within the specified ROI in injured explants compared with normal saline solution at all temperatures 4°C: 11.6% ± 0.2% (saline), 5.4% ± 0.4% (hyperosmolar); 21°C: 8.3% ± 0.4% (saline), 4.8% ± 0.2% (hyperosmolar); 37°C: 5.1% ± 0.3% (saline), 2.3% ± 0.1% (hyperosmolar) (**Fig. 2B**). Increasing the temperature of the normal saline solution from 4°C to 21°C resulted in 1.4-fold reduction in the PCD (*P* < 0.05) and warming to 37°C further reduced the PCD associated with scalpel-induced injury compared with 21°C (*P* < 0.05). The overall protection by raising temperature from 4°C to 37°C for saline irrigation was by approximately 2.3-fold (*P* < 0.001; **Fig. 2B**). Similarly, raising the temperature of the hyperosmolar saline solution to 37°C significantly decreased PCD by 2.4-fold compared with 4°C (*P* < 0.05 by ANOVA) (**Fig. 2B**). It should be noted that in control (uninjured) cartilage explants, there was no significant effect of either the normal saline solution or hyperosmotic saline solution on chondrocyte viability and no significant difference between these solutions (**Fig. 2B**). Thus overall, the use of the hyperosmotic irrigating solution at 37°C in the *in vitro* injury model decreased the PCD by about 5-fold compared with the PCD present with saline irrigation solution at 4°C.

### The Chondroprotective Effect of Hyperosmolar Saline Solution and Increased Temperature in an In Vivo Model of Cartilage Injury

**Figure 3.**
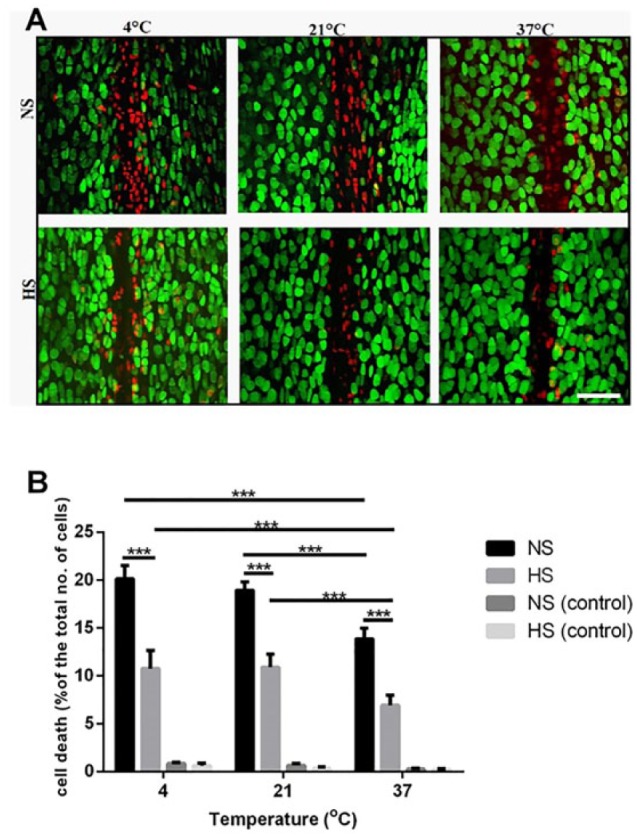
The chondroprotective effect of hyperosmotic saline solution and increased irrigation temperature in an *in vivo* model of scalpel-induced cartilage injury (**A**) Axial confocal laser scanning microscopy (CLSM) projection images of fluorescently labeled chondrocytes in injured cartilage exposed to normal saline solution (NS) or hyperosmolar saline solution (HS) at the indicated temperatures. Scale bar = 50µm. (**B**) The percentage of cell death in injured and uninjured control joints irrigated with hyperosmolar saline solution or normal saline solution at 4°C, 21°C, or 37°C. Data are expressed as mean ± SEM, [6(12)] for each data set. *P < 0.05, **P < 0.01, ***P < 0.001.

The PCD in the specified ROI used for the *in vivo* sham-operated joints irrigated at different osmotic pressures and temperatures was negligible (PCD = 0.45% ± 0.12% [6(36)]) under all conditions. Analysis of the CLSM images of injured cartilage showed approximately 50% reduction of chondrocyte death in joints irrigated with hyperosmolar saline solution compared with normal saline solution at all temperatures [4°C: 20% ± 0.4% (saline), 10.7% ± 1.9% (hyperosmolar saline solution); 21°C: 19% ± 0.9% (saline), 11% ± 0.3% (hyperosmolar saline solution); 37°C: 14% ± 1.1% (saline), 7% ± 1.0% (hyperosmolar saline solution) (*P* < 0.001 for each pair) (**Fig. 3**)]. Irrigation of the joints prior to and after injury with warm (37°C) saline solution significantly reduced cell death by ~30% compared with cold saline solution (4°C) or normal saline kept at 21°C (*P* < 0.001) (**Fig. 3B**).

Raising the temperature of the hyperosmolar saline solution to 37°C also significantly decreased the PCD in injured joints compared with this solution maintained at either 4°C (*P* < 0.001) or 21°C (*P* < 0.001) (**Fig. 3B**). It is notable that in control (uninjured) *in vivo* cartilage, there was no significant effect of either the normal saline solution or hyperosmotic saline solution on chondrocyte viability and no significant difference between these solutions (**Fig. 2B**). In summary, in this *in vivo* model of cartilage injury, the use of the hyperosmotic irrigating solution at 37°C decreased the PCD by approximately 3-fold compared with that present with saline irrigation solution at 4°C.

## Discussion

An isotonic NaCl saline solution at room temperature is commonly used for joint irrigation during orthopedic and arthroscopic procedures to provide a clear bloodless surgical field. However, this is likely to alter the temperature and extracellular osmolarity of *in situ* chondrocytes and may increase their sensitivity to mechanical trauma^8^ and adversely affect cartilage metabolism.^14,19^ Here, we investigated the effect of increasing osmolarity and temperature of a normal standard saline irrigation solution on chondrocyte viability using *in vitro* and *in vivo* models of cartilage injury. Irrigation with hyperosmolar saline solution significantly reduced cell death following scalpel-induced injury in both *in vitro* bovine and *in vivo* rat articular cartilage by approximately 50% at all temperatures examined. In addition, increasing the temperature of both irrigation solutions to 37°C significantly reduced cell death after mechanical injury. This suggests that in response to mechanical perturbation of articular cartilage, warm (37°C) hyperosmolar saline is chondroprotective compared with standard irrigation with normal saline at room (21°C) or cold (4°C) temperature.

In the *in vitro* experiments, a reproducible single linear cut was induced in osteochondral bovine cartilage explants using fresh scalpel blades. This minimized the variation in applied force and led to a consistent amount of cell death that was not correlated with minor variations in the width of the cut (**Fig. 1C** and **D**). This meant that the effect of altering solution temperature and osmolarity on cell death could be studied with greater sensitivity and reproducibility. Similarly, a validated *in vivo* model of scalpel-induced cartilage injury was chosen to represent partial thickness sharp mechanical trauma to articular cartilage.^9^

Irrigating healthy uninjured articular cartilage with either normal or hyperosmolar saline solution at 4°C, 21°C, or 37°C did not affect cell viability in either *in vitro* or *in vivo* models (**Figs. 2B** and **3B**). However, following sharp mechanical trauma to bovine and rat articular cartilage, chondrocyte death was significantly influenced by both changes in the osmolarity and temperature of the irrigation solution. The PCD present in the hyperosmolar solution at 37°C could be reduced by over 5-fold *in vitro* (**Fig. 2B**) and by approximately 3-fold *in vivo* (**Fig. 3B**) compared with a normal saline irrigation at 4°C. For the ROI used for the *in vivo* experiments, it is estimated that approximately 20% of the joint’s cartilage surface was studied. The single scalpel injury occurring in the presence of normal saline at 4°C caused 20% cell death (i.e., 4% of the total joint surface) and this was reduced to 7% by hyperosmotic saline at 37°C (i.e., about 1.3% of the total joint surface).

It is important to note that although this study provided proof of principle that increasing the osmolarity and temperature of normal saline reduced chondrocyte death associated with scalpel-induced injury, it has certain limitations. We emphasise that the results obtained from our experimental model/procedures cannot be regarded as translational to those occurring during orthopedic surgery and additionally only a single outcome measure, that is, that of chondrocyte viability, was assessed. The cartilage was only briefly exposed to the different irrigation solutions to elucidate the immediate influence of varying osmolarity and temperatures on the extent of cell death following cartilage injury. Previous studies have established that chondrocytes sense and respond within 5 minutes.^18^ Thus, it is in the early phase following the addition of the chondroprotective solution possibly as a result of chondrocyte shrinkage^7,18^ that influences the extent of chondrocyte death following mechanical injury. Research using an *in vivo* animal model has shown that short-term exposure of articular cartilage to hyperosmolar solution (600 mOsm; 5 minutes before and 5 minutes after mechanical injury) influenced long-term chondrocyte viability and cartilage regeneration by matrix constituents.^9^ This significantly improved the cartilage repair score compared to normal saline (300 mOsm) irrigation and this is why this short-term exposure was used in our present work.

There have been relatively few studies on the effect of longer term exposure of intact articular cartilage to irrigation solutions of differing osmolarity and temperature, and the influence on other cartilage structural and biological properties, for example, gene expression, synthetic and catabolic activities remain to be evaluated. However, in a porcine osteochondral explant model, exposure to saline irrigation solution at room temperature had a detrimental effect on chondrocyte metabolism and RNA synthesis.^14^ In addition, a recent report using a canine shoulder arthroscopy model^20^ demonstrated no deleterious effect on chondrocyte viability or cartilage hydration after 2hrs of irrigation with a hyperosmolar saline (600 mOsm by raised NaCl). Clearly, further work on the influence of long-term exposure of chondroprotective solutions on cartilage properties, extracellular matrix metabolism, and chondrocyte viability is warranted.

It is likely that unless care is taken, normal joint temperature will be reduced during articular surgery as a result of irrigation solution temperature, the length of the surgical procedure and exposure to the ambient conditions of the operating theatre. For example, it has been demonstrated that intra-articular temperature decreases from ~35°C at the beginning of anterior cruciate ligament (ACL) reconstruction or menisectomy surgery to ~25°C toward the end of the procedure after about 1.5hrs.^21^ These changes might have detrimental effects on the structural, physiological and biomechanical properties of articular cartilage, and potentially increase the sensitivity of chondrocytes to mechanical injury. In addition, exposure of articular cartilage to cold saline significantly altered the ultrastructure of the articular surface.^22^ During thermal chondroplasty, the temperature of the lavage solution influenced chondrocyte viability and surface contouring.^22^ The use of cool lavage solution (22°C) led to a significant increase in the depth of chondrocyte death and roughening of articular surface compared to a 37°C solution.^22^ Thus, the use of warmed, rather than cool, irrigation solutions has the potential to limit these deleterious effects if used for joint lavage.

The mechanism underlying the chondroprotective effect of warmed hyperosmolar saline against mechanical injury is unclear. This could be due to changes in matrix mechanics and/or direct effects on chondrocytes or chondrocyte-matrix interactions rendering them less sensitive to scalpel-induced injury. Previous work has demonstrated that raised temperature increased the dynamic and equilibrium stiffness of cartilage^23^ and therefore at 37°C the extracellular matrix at the injury edge could be stiffer and more resistant to disruption when injured by the scalpel. Although raising osmolarity might have a similar effect, the injury width was identical for scalpel-induced trauma with both saline and hyperosmotic saline irrigation over the temperature range studied (**Fig. 1C**). It is probable that a more direct action of temperature and osmolarity on chondrocytes or chondrocyte-matrix interactions are more important than their effects on bulk properties of cartilage. Changes to osmolarity affect the sensitivity and viability of chondrocytes following exposure to injurious stimuli.^7-9^ Thus, raising the osmolarity surrounding cartilage will decrease chondrocyte volume,^18^ membrane transport activity^24^ and lead to cytoskeletal reorganization.^25^ Changes to chondrocyte volume can initiate intracellular signaling cascades, including regulation of gene expression,^25^ metabolic activity,^19^ and calcium concentration,^26^ with the latter potentially interacting with other pathways such as those that generate reactive oxygen species (ROS), which control chondrocyte viability.^27^ Reducing temperature also has a variety of effects directly on cells, including increased membrane viscosity and stability^28^ decreased permeability^28^ and partial disassembly of spindle microtubules and cortical microfilaments.^29,30^ It is notable that the chondrocyte death at the site of matrix disruption reported here (**Figs. 2** and **3**) is similar to that observed following impact injury where cell death was localized around the cartilage cracks and not present in impacted areas without cracks.^7,31^ Indirect effects of reduced temperature and osmolarity on cell viability on the interactions between matrix proteins, chondrocyte integrins, and cytoplasmic elements may also be crucial as they are known to be essential for chondrocyte survival.^32^

In the short-term experiments reported here, warmed hyperosmolar saline was chondroprotective following mechanical injury. However, the long-term protection of cartilage integrity, stimulation of wound healing and the quality of the repair tissue remains to be investigated. Inhibiting chondrocyte death at the wound edge reduces matrix loss and enhances cartilage integration.^33^ We have previously shown that hyperosmotic lavage at room temperature resulted in a more developed repair tissue *in vivo* after 8 weeks compared with normal saline^9^ when applied before and after injury. It is therefore conceivable that the extra protection provided by warm irrigation solution may lead to further improvement in cartilage repair and lateral integration. Although this study used clearly defined and reproducible models of scalpel-induced injury, it is important to note that the nature, magnitude, and direction of any mechanical injury occurring during animal or human orthopedic surgery may be more complex and variable, with both blunt and sharp trauma arising from the use of various surgical tools such as osteotomes, pins, and screws. Consequently, the level of chondroprotection provided by raised osmolarity and temperature of the irrigation solution might differ from that reported here. The safety of a hyperosmolar saline solution in joint irrigation has been previously assessed in an *in vivo* rat model^9^ with no deleterious effects on any of the synovial joint soft tissues, for example, muscles, synovium, ligaments and menisci^9^ beyond those observed for normal saline irrigation. Thus, while irrigation with warmed hyperosmolar saline is a relatively simple chondroprotective procedure without adverse effect, further studies are needed to determine whether this solution is appropriate to preserve chondrocyte viability and promote cartilage repair following the injurious stimuli induced by other surgical instruments when applied to *in vitro* models or *in vivo* surgery.

This study provided evidence that in our animal models, increasing the osmolarity and temperature of the saline irrigation solution significantly reduced the extent of chondrocyte death associated with scalpel-induced injury. Furthermore, both the saline irrigation solution and the hyperosmolar solution had no detectable effects on the viability of uninjured cartilage. The use of this relatively simple and cheap hyperosmotic irrigation solution at 37°C may provide an effective strategy for conferring chondroprotection against mechanical injury both *in vivo* and *in vitro*.
